# Review of genotyping methods for *Yersinia pestis* in Madagascar

**DOI:** 10.1371/journal.pntd.0012252

**Published:** 2024-06-27

**Authors:** Lovasoa Nomena Randriantseheno, Voahangy Andrianaivoarimanana, Javier Pizarro-Cerdá, David M. Wagner, Minoarisoa Rajerison

**Affiliations:** 1 Plague Unit, Institut Pasteur de Madagascar, Antananarivo, Madagascar; 2 Ecole doctorale Sciences de la Vie et de l’Environnement, Faculty of Sciences, University of Antananarivo, Antananarivo, Madagascar; 3 Institut Pasteur, Université Paris Cité, CNRS UMR6047, *Yersinia* Research Unit, Paris, France; 4 Institut Pasteur, French National Reference Laboratory ‘Plague & Other Yersiniosis’, WHO Collaborating Centre for Plague FRA-140, Paris, France; 5 The Pathogen and Microbiome Institute, Northern Arizona University, Flagstaff, Arizona, United States of America; University of Texas Medical Branch, UNITED STATES

## Abstract

**Background:**

Plague, a zoonotic disease caused by *Yersinia pestis*, was responsible for 3 historical human pandemics that killed millions of people. It remains endemic in rodent populations in Africa, Asia, North America, and South America but human plague is rare in most of these locations. However, human plague is still highly prevalent in Madagascar, which typically records a significant part of all annual global cases. This has afforded an opportunity to study contemporary human plague in detail using various typing methods for *Y*. *pestis*.

**Aim:**

This review aims to summarize the methods that have been used to type *Y*. *pestis* in Madagascar along with the major discoveries that have been made using these approaches.

**Methods:**

Pubmed and Google Scholar were used to search for the keywords: “typing *Yersinia pestis* Madagascar,” “evolution *Yersinia pestis* Madagascar,” and “diversity *Yersinia pestis* Madagascar.” Eleven publications were relevant to our topic and further information was retrieved from references cited in those publications.

**Results:**

The history of *Y*. *pestis* typing in Madagascar can be divided in 2 periods: the pre-genomics and genomics eras. During the pre-genomics era, ribotyping, direct observation of plasmid content and plasmid restriction fragment length polymorphisms (RFLP) were employed but only revealed a limited amount of diversity among Malagasy *Y*. *pestis* strains. Extensive diversity only started to be revealed in the genomics era with the use of clustered regularly interspaced palindromic repeats (CRISPR), multiple-locus variable number tandem repeats (VNTR) analysis (MLVA), and single-nucleotide polymorphisms (SNPs) discovered from whole genome sequences. These higher-resolution genotyping methods have made it possible to highlight the distribution and persistence of genotypes in the different plague foci of Madagascar (Mahajanga and the Central and Northern Highlands) by genotyping strains from the same locations across years, to detect transfers between foci, to date the emergence of genotypes, and even to document the transmission of antimicrobial resistant (AMR) strains during a pneumonic plague outbreak. Despite these discoveries, there still remain topics that deserve to be explored, such as the contribution of horizontal gene transfer to the evolution of Malagasy *Y*. *pestis* strains and the evolutionary history of *Y*. *pestis* in Madagascar.

**Conclusions:**

Genotyping of *Y*. *pestis* has yielded important insights on plague in Madagascar, particularly since the advent of whole-genome sequencing (WGS). These include a better understanding of plague persistence in the environment, antimicrobial AMR and multi-drug resistance in *Y*. *pestis*, and the person-to-person spread of pneumonic plague. Considering that human plague is still a significant public health threat in Madagascar, these insights can be useful for controlling and preventing human plague in Madagascar and elsewhere, and also are relevant for understanding the historical pandemics and the possible use of *Y*. *pestis* as a biological weapon.

## Author summary

Plague remains a major public health concern in Madagascar and has been since its introduction to the country in 1898. The scale and rapid spread of the 2017 pneumonic plague epidemic in urban settings in Madagascar reminds us of the relevance of improving our understanding of this disease as well as its monitoring. In this review, we revisit the studies conducted in Madagascar that utilized genotyping approaches to understand the evolution, geographic distribution, and emergence of *Y*. *pestis*, the etiologic agent of plague. These studies greatly contributed to the comprehension of the origin, the transmission and the dynamics of the circulation of plague in Madagascar, providing useful information for preparing for future plague outbreaks in Madagascar and elsewhere.

## Introduction

*Yersinia pestis*, the etiologic agent of plague, is a fearsome pathogen. It has been responsible for 3 major historical pandemics [1]. The first pandemic wreaked havoc between the 6th and 8th centuries. As for the second pandemic, almost one third of the European population in medieval times was decimated during the Black Death epidemic alone [2]. Since 1855, due to global, human-mediated spread during the third pandemic [3], plague is geographically widespread; it is still maintained in rodent populations in parts of Africa, Asia, North America, and South America. However, human plague is rare in most of these locations, including in Central Asia [4] where all 3 historical pandemics likely originated [5]. Together with the Democratic Republic of the Congo (DRC), Madagascar reported >95% of plague cases in the world during the last decade [6–8]. For Madagascar particularly, the number typically reaches several hundred suspected cases each year [9] and sometimes thousands [10]. Because Madagascar has a large number of bubonic plague cases every year and pneumonic plague—the form that can spread person-to-person—arises from bubonic plague [9], it is one of the few places where pneumonic plague outbreaks regularly occur [11–15]. Because there is significant human and environmental plague activity here, genotyping *Y*. *pestis* isolates and DNA present in complex samples from Madagascar has yielded important insights that likely would not be possible from other locations. These include a better understanding of long-term persistence of *Y*. *pestis* in the environment, as well as information on rare events such as antimicrobial resistance (AMR) and multidrug resistance (MDR) in *Y*. *pestis*, person-to-person transmission of pneumonic plague in outbreaks/epidemics, and even transmission of AMR *Y*. *pestis* during a pneumonic plague outbreak. The insights gained from studying and genotyping *Y*. *pestis* from these events have relevance for controlling and preventing current plague activity in Madagascar and elsewhere, but also for understanding the historical pandemics and the possible use of *Y*. *pestis* as a biological weapon.

Initially, the classification of *Y*. *pestis* was based on biochemical characteristics, leading to the definition of 3 main biovars: Antiqua (fermenting glycerol, reducing nitrate), Medievalis (fermenting glycerol, not reducing nitrate), and Orientalis (not fermenting glycerol, reducing nitrate) [16]. However, this initially useful approach lacks resolution and it was later determined that these 3 biovars do not correspond to distinct phylogenetic lineages [17]. It then became clear that other methods were needed to effectively study the diversity within the species. In addition, *Y*. *pestis* is a young and clonal pathogen with little overall diversity (the sequences of 5 housekeeping genes being completely identical for *Y*. *pestis* isolated worldwide, including Madagascar [18]), and this is especially the case in Madagascar, as all *Y*. *pestis* populations here likely originated from a single introduction of the 1.ORI molecular group during the third pandemic [3]. As techniques and technologies advanced, successive attempts to decipher *Y*. *pestis* genetic diversity in Madagascar can be divided into 2 main eras: the pre-genomics era and the genomics era. Here, we review all genotyping techniques that have been used in the context of Madagascar to classify *Y*. *pestis* and report the main results and findings from these studies.

## Methods

The review was conducted using Pubmed and Google Scholar; we searched for the following keywords: “typing *Yersinia pestis* Madagascar,” “evolution *Yersinia pestis* Madagascar,” and “diversity *Yersinia pestis* Madagascar.” Given the limited body of literature dedicated to this particular topic, we only found 11 publications relevant to the scope of this review. References cited in each of these papers were subsequently reviewed to find additional publications.

## Results and discussion

The history of *Y*. *pestis* genotyping in Madagascar can be divided into 2 periods: the pre-genomics and genomics eras (Table 1).

**Table 1:** Summary of the different advantages and drawbacks of the techniques used to genotype *Y*. *pestis* in Madagascar.

Era	Techniques	Advantages	Disadvantages
Pre-genomics era	Ribotyping	Provided initial discrimination among isolates at a time when no other approaches were available.	Laborious Requirement for isolation and manipulation of live strains Low resolution
	Direct observation of plasmid content and plasmid-RFLP	Identification of the loss of one or more virulence plasmid(s) Identification of the presence of new plasmids	Laborious Low resolution Requirement for isolation and manipulation of live strains
Genomics era	WGS	Able to discover new genetic determinants of phenotypic traits (e.g., plasmids, AMR genes, virulence genes) Reveals the comprehensive diversity of the strains studied	Expensive High-throughput sequencing platforms not yet available in Madagascar Requirement for isolation and manipulation of live strains to obtain genomic DNA extracts
	CRISPR	Correlation between genotypes and geographic locations Applicable to DNA extracted from complex clinical samples without the need for culture	Low resolution Sequencing not available in Madagascar and expensive The possible acquisition of a new spacer in an interstitial position and the loss of multiple spacers
	MLVA	High resolution Applicable to DNA extracted from complex clinical samples without the need for culture Able to trace transfers	High mutation rate making the technique susceptible to homoplasy and mutational saturation Not always possible to identify deeper phylogenetic relationships (major groups)
	SNPs	Low mutation rate (almost no homoplasy) Able to identify deep phylogenetic relationships (major groups) Applicable to DNA extracted from clinical samples without the need for culture Useful for dating phylogenetic trees Able to trace transfers	Low mutation rate (unable to resolve some closely related strains) but can be overcome somewhat by examining SNPs across the entire genome
	DNA capture + enrichment	Applicable to clinical samples with low level of target DNA without the need for culture Useful particularly when live strains are not available	Laborious Complex design and selection of RNA baits Cost of reagents (e.g., RNA baits, streptavidin-coated beads) Cost of sequencing

### Pre-genomics era

#### Ribotyping

Ribotyping consists of cutting the target DNA with restriction enzymes, running the fragments with gel electrophoresis, transferring the separated fragments into a membrane, and then hybridizing them with labeled probes that are complementary to the targeted ribosomal RNA (rRNA) gene sequences (Southern blotting). This technique results in the visualization of the profile (ribotype) of the DNA fragments containing rRNA genes. For *Y*. *pestis*, EcoRI and EcoRV restriction enzymes were used to fragment the DNA. It was first used to assess global *Y*. *pestis* diversity in 1994 [19], and in 1997 the diversity of *Y*. *pestis* strains from Madagascar was investigated using this technique [20]. Nineteen ribotypes were identified among global *Y*. *pestis* strains [19], with 4 of these found in Madagascar: B, Q, R, and T [20]. Of these, 3 (Q, R, and T) were only identified from Malagasy strains.

The Q, R, and T ribotypes appear to have emerged in different locations in the Central Highlands foci. The Q ribotype was first discovered in 1983 in 2 different locations: Ambovombe in Manandriana District and Vohiposa in Ambohimahasoa District. The R ribotype was first reported in 1982 from Andina in the Northern part of the Ambositra district, and the first strains of the T ribotype were detected in Talata Vohimena in Manandriana District in 1994 [20]. Studies of 131 strains isolated in Ambositra and Ambohimahasoa between 1940 and 1996 revealed 58.02% of B ribotype, 18.32% of R ribotype, 21.37% of Q ribotype, 1.53% of T ribotype, and 0.76% coinfection by R and Q ribotypes [20]. By investigating the correlation between each ribotype and case outcomes, a link between the R ribotype and serious symptoms resulting in fatal outcome was suggested [20] without further investigation. Because ribotyping requires isolation and manipulation of live strains, a very limited number of strains have been tested with this technique. It is not used anymore for this reason and its low level of resolution.

#### Direct observation of plasmid content and plasmid restriction fragment length polymorphisms (RFLP)

Most *Y*. *pestis* strains contain 3 plasmids: pPla/pPCP1 (approximately 10 kb), pCD1/pYV (approximately 70 kb), and pFra/pMT1 (approximately 100 kb) [21–26]; some contain even more [23,27–32]. The 3 main plasmids play an important role in virulence of *Y*. *pestis* because of the virulence factors encoded by them (e.g., type III secretion system [33], capsular antigen [34], and plasminogen activator [35]), and differences regarding their presence have been investigated by examining plasmid content directly or by observing plasmid restriction profiles. These methods consist, respectively, of extracting plasmids from strains and analyzing them directly with electrophoresis [23], or subjecting them to the EcoRV restriction enzyme to generate fragments of different sizes prior to analysis with gel electrophoresis [20,36,37]. Only 1 strain from Madagascar (vaccine EV strain) has been tested using direct observation of plasmid content and the size of its pCD1 plasmid (45 MDa) was determined to be similar to that of other 1.ORI strains, but smaller than that of strains from other countries (47 MDa, 48 MDa, and 49 MDa) [23]; no additional plasmids were detected in this strain. Using plasmid RFLP, 5 *Y*. *pestis* strains from Madagascar have been reported to have lost at least one of the main plasmids as evidenced by the absence of some fragments compared to a reference *Y*. *pestis* strain (strain 6/69) [20], with the specific identification of the lost plasmid(s) having not been the subject of further study. On the other hand, additional bands present in the profile of 15 other Malagasy strains [20,36,37] suggested the presence of supplementary plasmids, which has been confirmed in only 2 isolates (16/95 and 17/95). These novel plasmids conferred resistance to streptomycin for 16/95 [37] and to multiple antibiotics (ampicillin, chloramphenicol, kanamycin, streptomycin, spectinomycin, sulfonamides, tetracycline, minocycline) for 17/95 [36]. The drawbacks of direct observation of plasmid content and plasmid-RFLP are their lack of resolution and the need to isolate and manipulate live strains.

### Genomics era

#### Whole-genome sequencing (WGS)

Since the first *Y*. *pestis* genome became available in 2001 [38], it has been possible to compare multiple *Y*. *pestis* genomes. In Madagascar, WGS has been used mainly to identify genetic markers of evolution, such as single-nucleotide polymorphisms (SNPs), in order to use them to genotype strains that have not been sequenced [3,39,40]. Increasingly, all the strains in a study are sequenced, with the resulting whole genome sequences used to directly call SNPs and to infer the phylogeny [41,42]. In that case, the genotyping process is more exhaustive because it captures the diversity of the entire genome sequence of all the strains studied. This technique has also been used to identify genetic determinants that could support new phenotypic characteristics, such as antibiotic resistance [11]. Indeed, 3 AMR strains from Madagascar [11] acquired resistance to streptomycin via the same spontaneous point mutation in the 30S ribosomal protein S12 (*rpsL*) gene, thereby documenting that AMR can also arise in *Y*. *pestis* via point mutations. Two of these strains were isolated from the same pneumonic plague outbreak and were 100% identical with WGS, also documenting for the first time that AMR strains can spread person-to-person via primary pneumonic plague [11], which is a possible scenario that might occur if *Y*. *pestis* was utilized as a bioweapon [43]. More recently, WGS of *Y*. *pestis* isolates was used to characterize local plague activity in multiple endemic rural foci from August to November 2017 [44], with subsequent phylogenetic analyses assigning isolates to the same distinct *Y*. *pestis* lineages that have been persisting in those different foci for many years [39]. Overall, this technique has the advantage to reveal the diversity of the strains included in a study in their entirety. However, the downside is that sequencing all the strains in a given study is still quite expensive, whether for reagents and equipment, or for shipping samples elsewhere for sequencing. This is why in most of the studies undertaken in Madagascar until now, only a subset of the strains included in the studies have been sequenced.

#### Clustered regularly interspaced palindromic repeats (CRISPR)

A CRISPR locus is a genetic structure made up of conserved repeat regions separated by unrepeated elements called spacers. A 300 to 500 bp sequence called the “leader” is adjacent to and located at the end of CRISPR loci. The repeated sequences as well as the leader sequence are conserved within a bacterial species and are different between 2 distinct species [45]. The existence of 3 CRISPR loci—YP1, YP2, and YP3 (sometimes also referred to as loci A, B, and C, respectively)—with the same 28 bp repeat sequence in the *Y*. *pestis* genome was first described in 2002 [45]. For YP1 and YP2, the first repeat is truncated (19 bp for YP1 and 22 bp for YP2) [46]. The study of CRISPR in *Y*. *pestis* led to the formulation of 3 rules for the evolution of CRISPR loci [46], which were then subsequently used to study the evolution of the strains carrying them. First, deletion of one or more spacers can occur and is random. Second, it has been shown that the acquisition of new spacers occurs in a polarized fashion and is only on one side (between the leader sequence and the last repeated sequence), but before that, the repeated sequence directly upstream of the leader sequence is first duplicated and added just in front of the leader sequence. Third, the occurrence of the same spacers in a CRISPR allele indicates a common ancestral origin rather than arising from independent events.

For the study of CRISPR in *Y*. *pestis*, 2 different aspects of the same spacer nomenclature exist. The first consists of assigning a letter to each spacer according to the order of discovery. For YP2 and YP3 loci, the number 2 or 3 is added to the letter, respectively [46]. Each combination of spacers corresponds to an allele (for example, the allele of YP1 locus of *Y*. *pestis* strain CO92 is: abcdefgh). In 2005, based on this nomenclature, Pourcel and colleagues identified and named twenty-one, nine, and three alleles for YP1, YP2, and YP3, respectively, in *Y*. *pestis* strains isolated worldwide, and also reported the first CRISPR genotyping of Malagasy strains [46]. The second aspect consists in adding a number after a, b, or c depending on the locus considered (A, B, or C, respectively). Because letters are in finite number (26, a–z), this second aspect of the same nomenclature was used more often as new spacers continued to be described [47]. Seventeen other spacers (13 for locus A, 2 for locus B, and 2 for locus C) that appear to be specific to Madagascar were discovered during the analyses performed on clinical samples from plague patients during the 2007 plague season [48] and named using this nomenclature: a89, a90, a91, a92, a93, a94, a95, a96, a97, a98, a99, a100, a102 for locus A spacers; b5′ and b50 for locus B spacers; and c11 and c13 for locus C spacers. Taken together, the alleles of YP1, YP2, and YP3 constitute a genotype.

One advantage of CRISPR is in the correlation found between CRISPR genotypes/spacers and geographic regions, helping to discern population structures and phylogeography of *Y*. *pestis* [49]. Another advantage is that, unlike the above genotyping methods, CRISPR genotyping can be performed directly on DNA extracted from complex biological samples without the need to isolate a strain [48,50]. This method was therefore used to genotype *Y*. *pestis*-positive DNA extracts obtained from human and animal biological samples during the investigation of the 2011 pneumonic plague outbreak in the Ambilobe district (an area outside the known plague foci located in northern Madagascar) during which a new Madagascar-specific spacer of the YP3 locus was discovered at 2 different locations separated by around 400 km (Ambarakaraka in Ambilobe district and Bealanana in Bealanana district) [50]. Based on the discovery of 4 CRISPR genotypes circulating locally, it was concluded that Ambilobe was a natural plague focus long before 2011 [50]. The most frequent genotype, assumed to have caused the outbreak, was also found in Ankazobe, suggesting anterior expansion from the Central Highlands [50]. However, the disadvantages to this technique are its low discriminatory power, the cost of sequencing for spacers identification, and the possible exceptions to the polarized acquisition of spacers as described in other bacteria [51], thereby leading to the failure of CRISPR to properly reflect evolution.

#### Multiple-locus variable number tandem repeats (VNTR) analysis (MLVA)

MLVA, as indicated by its name, implies the analysis of multiple VNTR loci. VNTRs, also called minisatellites or microsatellites depending on the number of nucleotide(s) repeated, are tandem repeated sequences in which variability at a given VNTR locus concerns the number of repeats that differ among strains. Therefore, genotyping with this method consists in determining the number of repetitions at each locus for each strain, each number representing an allele. Consequently, a VNTR locus can have many different alleles. Starting in 2011, a hierarchical SNPs-MLVA system containing 43 VNTR loci identified from the early *Y*. *pestis* genomes [52–54] emerged as the pioneering technique to unveil the extensive diversity exhibited by *Y*. *pestis* in Madagascar, successfully identifying a total of 226 genotypes from the 262 examined strains [55].

MLVA genotyping is useful because, like CRISPR, isolation of *Y*. *pestis* strains is not mandatory, it can be performed with DNA extracted from different types of human and environmental samples [48]. The high mutation rate of VNTRs explains the discriminatory nature of MLVA, enabling different strains of a clonal species such as *Y*. *pestis* to be distinguished. It was also the first technique that allowed an extensive phylogeographic study of *Y*. *pestis* in Madagascar, providing insights into the distribution of different genetic groups across years and throughout the different plague foci [55]. Many discoveries have been made using this technique. Among them, the identification of 11 and 4 subclades, respectively, in major phylogenetic groups I (I.A-I.K) and II (II.A-II.D). These 15 subclades appeared to be geographically separated [55], suggesting that a specific genotype was initially introduced into a given area with subsequent local differentiation (founder effect). The previous studies on these MLVA subgroups concluded that strains assigning to group I were found across all the Malagasy plague foci (Central and Northern Highlands and Mahajanga), whereas strains assigning to group II were only found in the Central Highlands in plague hotspots such as the Betafo, Manandriana, Ambositra, and Ambatofinandrahana districts [55]. Within group I, subclades found in the Northern Highlands foci (I.C, I.G, and I.I) are different from those found in the Central Highlands (I.A, I.B, I.D, I.E, I.F, I.H, I.J, and I.K). Regarding the coastal focus of Mahajanga, *Y*. *pestis* found there is dominated by the I.A subclade, even forming a subcluster called the Mahajanga I.A subclade [55]. This high fidelity between geographic locations and specific subpopulations of *Y*. *pestis* means that genetic typing can be used to identify rare transfer events, such as multiple introductions of *Y*. *pestis* from the Central Highlands to the coastal city of Madagascar, where it persisted for some years before eventually dying out [40,41]. Finally, Ambositra was among the most diverse districts in terms of *Y*. *pestis* genotypes, with 6 MLVA subclades isolated from there. Based on this finding, it was suggested as the district of origin for the I.A subclade as the earliest strains assigned to this subclade to date were isolated there [55].

The high mutation rate of VNTRs is both a strength and a weakness of these markers. A strength because of the high resolution it provides among even very closely related strains, but also a weakness because, in some cases, its inherent high mutation rate may lead to mutational saturation and homoplasy distorting the reconstruction of the phylogeny [56] or yielding low bootstraps in phylogenies [55]. Mutational saturation is the state reached when additional mutations do not bring additional genetic diversity to the locus anymore meaning that studying that locus does not give comprehensive information on the evolution of the species because some mutations are not detectable and balanced out by recurrent mutations [56]. Due to these phenomena, MLVA also sometimes fails to identify deeper relations like those between the 2 major groups [55], and mutations in VNTRs loci may even happen during laboratory cultivation making the strains studied not completely identical to the original ones [57]. To overcome these problems, a hierarchical approach is often adopted: SNPs are used first because they have lower mutation rates and so can resolve the major groups, and then, MLVA is used to split strains assigned to these major groups into subgroups because of their higher resolution that can discriminate more closely related strains.

#### Single-nucleotide polymorphisms (SNPs)

SNPs are substitutions involving only a single nucleotide and, in general, *Y*. *pestis* genotyping using SNPs can be done in 2 different ways. The first consists in determining the allele carried by each strain studied by amplification with specific primers (polymerase chain reaction or PCR) [58]. This first technique can only be applied to SNPs already described [58] or newly described following the whole genome sequence comparisons of a subset of strains [39,40,55] and, in general, is not exhaustive in the sense that if other evolutionary informative SNPs are present in the strains not sequenced, those will not be considered during genotyping, creating what is called a “discovery bias” [59]. The second technique consists in WGS of each of the strains included in the study as reported above; however, a bias persists given that sequencing all the strains existing in Madagascar is often practically impossible.

SNPs genotyping has been particularly important in the case of *Y*. *pestis* in Madagascar because it is the only technique capable of reliably distinguishing the 2 major groups (group I and group II) circulating in the country [3] differentiated by the Mad-43 allele [position 1,348,724 in the *Y*. *pestis* strain CO92 genome (accession NC_003143.1), ancestral state (C) for group I and derived state (T) for group II] [55], and one of the techniques that really revealed the extensive diversity of *Y*. *pestis* in Madagascar. Indeed, new subgroups (y, z, ɑ, and β in the group I; t, u, v, w, and x in the group II) and new nodes within different subgroups could be identified with the identification of new SNPs as more strains were whole-genome sequenced [39,44]. This higher level of resolution allowed the subsequent studies of the distribution of populations across foci. Genotyping *Y*. *pestis* isolates obtained from the same locations across multiple years provided insights into plague persistence in the environment, including identification of the specific *Y*. *pestis* subpopulation that persists in a given location, and this background information is important for determining the likely geographic origins and spread of plague activity in humans. Although multiple hypotheses have been proposed to explain it (reviewed in [60,61]), plague persistence in the environment is still poorly understood in most global regions where it occurs. In Madagascar, plague is highly endemic in rodent populations in rural foci throughout the Central and Northern Highland [62], leading to regular spillover into humans [9], so it was possible to use MLVA and SNPs to genotype >750 *Y*. *pestis* isolates collected across multiple years from the same rural foci [39]. The results revealed that distinct *Y*. *pestis* subpopulations are found in different rural foci in the highlands and persist in those locations across years, documenting that—at least in Madagascar—plague persists locally in rat and flea populations [62]. And again, the invaluable insights into the phylogeography of *Y*. *pestis* in Madagascar provided by these studies enabled the detection of transfers with their directionality as described for the s subgroup [40]. It is thought to have emerged in the Ambositra district where s1 strains (the most ancestral node within s subgroup) were found and then spread successively to other districts: Soavinandriana (s2 and s3), Miarinarivo (s3 and s4), Arivonimamo (s3), Antananarivo (s3, s5, s8) and Manjakandriana (s3 and s5), Antanifotsy (s5), Fianarantsoa (s5), and Mahajanga (s5, s8 and s9) [40]. Following studies also found that MLVA and SNP subgroups were highly congruent (7 out of the 13 MLVA subgroups in group I showed congruence with SNP subgroups and 6 out of the 7 for group II [39]), making it possible to only screen SNPs for these congruent subgroups, which is easier. It can also be applied to clinical samples [48,50], although it has been reported to have lower discriminatory power compared to MLVA or CRISPR in a previous study [48]. A summary of the distribution of genetic diversity of *Y*. *pestis* (according to isolation time range and geographical location) in Madagascar using MLVA and SNPs techniques is provided in Table 2 below.

**Table 2:** Distribution of genetic diversity of *Y*. *pestis* in Madagascar.

MLVAID	SNP ID	Years covered by the study	Number of subtypes	Number of strains	Number of complex samples	Region	Districts	Citation
I.A	s	1939–2005	9*	89	0	Boeny	Mahajanga	[40,55]
						Analamanga	Antananarivo, Manjakandriana	
						Itasy	Arivonimamo, Miarinarivo, Soavinandriana	
						Vakinankaratra	Antanifotsy	
						Amoron’i Mania	Ambositra	
						Haute Matsiatra	Fianarantsoa	
		1995–2012	22	157	31	Boeny	Mahajanga I	[39]
						Alaotra Mangoro	Moramanga, Anosibe An’Ala	
						Analamanga	Antananarivo Avaradrano, Antananarivo Renivohitra, Anjozorobe, Ankazobe, Manjakandriana	
						Bongolava	Fenoarivobe, Tsiroanomandidy	
						Itasy	Arivonimamo, Miarinarivo, Soavinandriana	
						Vakinankaratra	Antanifotsy, Mandoto, Betafo, Faratsiho, Antsirabe II	
						Amoron’i Mania	Ambatofinandrahana, Fandriana, Ambositra	
						Haute Matsiatra	Isandra, Vohibato	
		1995–2017	22	41	0	Boeny	Mahajanga I	[44]
						Analamanga	Andramasina, Antananarivo Avaradrano, Ankazobe, Manjakandriana	
						Bongolava	Tsiroanomandidy	
						Itasy	Miarinarivo, Arivonimamo	
						Vakinankaratra	Faratsiho, Betafo	
I.B	q	1939–2005	7	12	0	Alaotra Mangoro	Moramanga, Ambatondrazaka	[40,55]
						Analamanga	Antananarivo, Anjozorobe, Manjakandriana	
		1995–2012	14	87	14	Alaotra Mangoro	Amparafaravola, Moramanga, Andilamena,	[39]
						Analamanga	Antananarivo Renivohitra, Anjozorobe, Manjakandriana	
						Betsiboka	Tsaratanana	
						Bongolava	Fenoarivobe	
						Amoron’i Mania	Ambatofinandrahana	
						Vakinankaratra	Antsirabe II, Betafo	
		1995–2017	15	7	0	Alaotra Mangoro	Moramanga	[44]
						Analamanga	Antananarivo Avaradrano, Manjakandriana	
						Vakinankaratra	Betafo	
I.C		1939–2005	-	2	0	SOFIA	Bealanana	[39,55]
I.D	y	1939–2005	-	17	0	Amoron’i Mania	Manandriana, Ambatofinandrahana	[55]
						Haute Matsiatra	Ambohimahasoa	
		1995–2012	5	10	2	Amoron’i Mania	Ambositra, Manandriana, Ambatofinandrahana	[39]
						Haute Matsiatra	Ambohimahasoa	
		1995–2017	5	3	0	Amoron’i Mania	Ambositra, Manandriana, Ambatofinandrahana	[44]
I.E		1939–2005	-	11	0	Analamanga	Antananarivo	[55]
						Amoron’i Mania	Ambositra	
						Haute Matsiatra	Fianarantsoa	
						Fitovinany	Ikongo	
		1995–2012	-	12	1	Boeny	Mahajanga I	[39]
						Analamanga	Antananarivo Renivohitra	
						Amoron’i Mania	Ambositra	
						Haute Matsiatra	Vohibato, Isandra	
						Fitovinany	Ikongo	
I.F	r	1939–2005	-	6	0	Boeny	Mahajanga	[40,55]
						Analamanga	Antananarivo	
						Amoron’i Mania	Manandriana	
						Haute Matsiatra	Fianarantsoa, Ambohimahasoa	
		1995–2012	-	5	0	Alaotra Mangoro	Moramanga	[39]
						Vakinankaratra	Antsirabe II	
						Haute Matsiatra	Ambohimahasoa, Isandra	
I.G		1995–2012	-	4	0	SOFIA	Bealanana	[39,55]
I.H	l	1939–2005	2	2	0	Haute Matsiatra	Fianarantsoa, Ambalavao	[40,55]
		1995–2012	2	3	2	Vakinankaratra	Antsirabe II	[39]
						Haute Matsiatra	Isandra, Ambalavao	
		1995–2017	2	1	0	Haute Matsiatra	Ambalavao	[44]
I.I		1939–2005	-	3	0	SOFIA	Bealanana	[55]
I.J	j	1939–2005	-	6	0	Bongolava	Tsiroanomandidy	[40,55]
						Itasy	Soavinandriana	
						Vakinankaratra	Betafo	
		1995–2012	10	91	5	Analamanga	Anjozorobe	[39]
						Bongolava	Tsiroanomandidy	
						Itasy	Soavinandriana	
						Vakinankaratra	Betafo, Faratsiho, Mandoto, Antsirabe I, Antsirabe II	
		1995–2017	10	13	0	Analamanga	Antananarivo Renivohitra	[44]
						Itasy	Soavinandriana	
						Atsinanana	Brickaville	
						Vakinankaratra	Betafo, Mandoto	
						Bongolava	Tsiroanomandidy	
I.K		1939–2005	-	8	0	Analamanga	Antananarivo, Ankazobe	[55]
						Itasy	Miarinarivo	
						Vakinankaratra	Antsirabe	
						Amorin’i Mania	Ambositra	
I.L		1995–2012	-	0	17	Haute Matsiatra	Ambalavao	[39]
I.M	z	1995–2012	6	13	0	Boeny	Mahajanga I	[39]
						Vakinankaratra	Antanifotsy, Antsirabe II, Betafo	
						Amoron’i Mania	Fandriana	
		1995–2017	6	1	0	Vakinankaratra	Antanifotsy	[44]
II.A	t	1939–2005	-	32	0	Analamanga	Antananarivo	[55]
						Amoron’i Mania	Ambositra, Ambatofinandrahana	
						Haute Matsiatra	Manandriana	
		1995–2012	11	45	12	Analamanga	Anjozorobe	[39]
						Vakinankaratra	Antsirabe II, Betafo	
						Amoron’i Mania	Ambatofinandrahana, Ambositra, Fandriana, Manandriana	
		1995–2017	11	3	0	Vakinankaratra	Antsirabe II	[44]
						Amoron’i Mania	Manandriana	
II.B	h	1939–2005	2	24	0	Vakinankaratra	Betafo	[40,55]
						Amoron’i Mania	Ambositra, Ambatofinandrahana	
		1995–2012	16	101	0	Vakinankaratra	Betafo, Antsirabe I, Antsirabe II, Mandoto	[39]
						Amoron’i Mania	Ambositra, Ambatofinandrahana	
		1995–2017	16	4	0	Vakinankaratra	Antsirabe II, Betafo	[44]
						Amoron’i Mania	Ambatofinandrahana	
II.C	v	1939–2005	-	5	0	Vakinankaratra	Betafo	[55]
						Amoron’i Mania	Ambositra	
		1995–2012	13	116	0	Vakinankaratra	Betafo, Antsirabe II, Mandoto	[39]
						Amoron’i Mania	Fandriana	
		1995–2017	13	5	0	Vakinankaratra	Antsirabe II, Betafo, Mandoto	[44]
II.D.1			-	13	0	Amoron’i Mania	Ambatofinandrahana	[39,55]
II.D.2	w	1995–2012	2	19	0	Vakinankaratra	Antsirabe II	[39]
		1995–2017	2	1	0	Vakinankaratra	Antsirabe II	[44]
II.E	u	1995–2012	3	5	1	Amoron’i Mania	Fandriana	[39]
		1995–2017	3	1	0	Amoron’i Mania	Fandriana	[44]
II.F	x	1995–2012	3	6	0	Alaotra Mangoro	Moramanga	[39]
						Itasy	Arivonimamo, Miarinarivo	
						Vakinankaratra	Faratsiho	
		1995–2017	3	3	0	Itasy	Arivonimamo	[44]
						Vakinankaratra	Faratsiho	
	e	1939–2005	-	1	0	Vakinankaratra	Antanifotsy	[40,55]
	ɑ	1995–2017	-	1	0	SOFIA	Mandritsara	[44]
	β	1995–2017	-	6	0	Analamanga	Antananarivo Avaradrano	[44]
						Haute Matsiatra	Ambalavao	
	k	1939–2005	-	21	0	SOFIA	Mandritsara, Bealanana	[55]
						Boeny	Mahajanga	
						Analamanga	Antananarivo	
						Bongolava	Tsiroanomandidy	
						Itasy	Arivonimamo, Soavinandriana	
						Vakinankaratra	Ambatolampy, Antsirabe, Betafo	
						Amoron’i Mania	Ambositra	
						Haute Matsiatra	Fianarantsoa, Ambohimahasoa	
		1995–2012	-	3	0	Alaotra Mangoro	Moramanga	[39]
						Vakinankaratra	Antsirabe II	
	d	1939–2005	-	3	0	Itasy	Soavinandriana, Miarinarivo	[55]
		1995–2012	-	1	0	Vakinankaratra	Antsirabe II	[39]
	i	1939–2005	-	1	0	Amoron’i Mania	Fandriana	[55]
	o	1939–2005	-	1	0	Bongolava	Tsiroanomandidy	[55]
	p	1939–2005	-	1	0	Vakinankaratra	Antanifotsy	[55]

*The number of subtypes has only been determined in [40].

Finally, dating a phylogenetic tree generated using SNPs is possible when metadata, such as date of strain isolation, is available, and results can provide new insights into the epidemiology of the infectious disease of interest. Recent studies have focused on dating phylogenetic trees of *Y*. *pestis* to determine the divergence times of the different clades [39,41]. The authors studied the transmission events between Madagascar and other countries (from India to Madagascar, from Madagascar to Turkey) and inside Madagascar (between the Central Highlands and Mahajanga) [41]. For inter-countries transmissions, the median tMRCA (the time to the most recent common ancestor) of 1.ORI2a (Indian strains) and 1.ORI3 (Malagasy strains) is estimated at 1877 meaning that these 2 clades probably separated around that time [41]. Regarding the transmission event to Turkey, the tMRCA of the 1.ORI3 strains from Turkey and Madagascar has been estimated to be 1905 [41]. As 1.ORI3 is present exclusively in Madagascar and Turkey, the authors emitted the hypothesis that the lineage first introduced into the island was 1.ORI2a, and then 1.ORI3 emerged probably following transmissions and adaptation to local reservoirs and subsequently spread to Turkey from Madagascar [41]. Regarding transmissions between different foci in Madagascar, 5 transfers have been identified between the Central Highlands and Mahajanga, confirming the hypothesis formulated earlier based on the study of MLVA [40]; four of them from the Central Highlands to Mahajanga and 2 out of the 4 would have been responsible of the 1990s epidemics [41]. These findings also reinforced the hypothesis of a reintroduction as a source of plague in Mahajanga rather than a reemergence [40,41].

#### Whole-genome DNA capture and enrichment

Whole-genome DNA capture and enrichment techniques are typically used to amplify low level DNA signals before sequencing. They consist in first designing biotinylated probes targeting the organism of interest using a reference genome, extracting the DNA from the samples, building libraries, hybridizing the libraries with the probes, capturing the hybridized libraries-probes with streptavidin-coated beads, and then sequencing [63]. This technique was extensively used in ancient DNA studies [63–65] but is increasingly used in contemporary studies using complex clinical samples as starting point. This takes advantage of the technique’s ability to specifically capture the target genome and increase the signal in samples composed of DNA from multiple origins, thereby increasing the probability of detection of the pathogen of interest. In the case of plague in Madagascar, the first description of the use of this technique was in the recent study of the 2017 urban pneumonic plague epidemic [44] for which only few *Y*. *pestis* strains were available. It was of key importance to determine that the epidemic was caused by multiple introductions of *Y*. *pestis* from several different rural foci via travel of infected individuals into urban areas [44]. The main drawback of the technique is its cost.

### Limitations of previous studies and current unknowns

#### Discovery bias: We could only find what we were looking for

WGS is a relatively new technique that is still expensive, particularly for a low-income country like Madagascar. This, combined with the fact that to date it has only been available outside Madagascar, led to only a limited number of *Y*. *pestis* isolates from Madagascar being entirely sequenced and serving as a basis to the discovery of informative SNPs. The SNPs discovered from a limited set of whole-genome sequences was then used to infer phylogeographic patterns within a larger set of strains. This method is very powerful and cost-effective compared to sequencing all strains included in a study. However, a problem remains: the identified SNPs represent the diversity of the strains sequenced only, not the entire set of strains (nor the entire collection) therefore, if other SNPs exist in any of the strains that have not been sequenced, they will not be included in the reconstruction of the phylogeny, this phenomenon is called “discovery bias” [59]. The consequence of that bias is primarily in the topology of the resulting tree, which would have some branches collapsed, but also an underestimation of the real diversity of the strains [59]. This could be rectified by establishing the capability to regularly perform WGS of all new strains collected in Madagascar, as well as archived strains.

#### The unknown contribution of horizontal gene transfer (HGT) to the local evolution of *Y*. *pestis* in Madagascar

Recent studies on the evolution of *Y*. *pestis* in Madagascar focused mainly on SNPs and MLVA techniques. These techniques are very reliable and allowed to answer important questions, particularly the origin of the outbreaks in Mahajanga [40,41], the distribution of *Y*. *pestis* genotypes across different plague foci, and their local evolution [39,55,66]. However, one aspect of the evolution cannot be grasped by these approaches, namely HGT. HGT is a very important aspect of evolution in bacteria, particularly for the members of Enterobacteriaceae family and even in eukaryotes [67], because it allows the acquisition of new features, such as novel virulence mechanisms, resistance to antibiotics, resistance to biocides. Three mechanisms of HGT are known in bacteria: conjugation, transformation, and transduction.

Although plasmid genotyping, as we mentioned earlier in this review, has initiated investigations into the difference in plasmid content of different strains (conjugation), such research was not subsequently pursued. Even though *Y*. *pestis* has been considered clonal and HGT via conjugation is rare in it, this type of transfer has still been documented, and was even at the origin of the species itself when it acquired its two novel virulence plasmids, pPla/pPCP1 and pFra/pMT1. HGT has also allowed some *Y*. *pestis* strains in Madagascar to acquire plasmids with resistance genes to one (streptomycin or doxycyclin) [37,68] or multiple antibiotics [36]. Other features acquired through HGT have also been described in *Y*. *pestis* isolated in other countries, such as arsenic resistance in Java 9 strain [29]. YpfФ is a prophage of 8.7 kb [69], most likely acquired through HGT also, that has been described in the chromosome of *Y*. *pestis* and reported to affect the fitness and the pathogenicity of *Y*. *pestis* [70]. Therefore, HGT is important in the evolution of *Y*. *pestis* and deserves to be better investigated in future research.

## Conclusions

Genotyping *Y*. *pestis* has always been challenging because of its limited diversity due to its recent emergence as a species and clonal structure. However, genotyping is important to study plague epidemiology and to monitor the evolution of the plague bacterium in order to have a better insight on how it might evolve in the future and to know if it might acquire (or have already acquired) new features that would make the disease harder to control such as AMR/MDR, new virulence factors, new genes that change the symptomatology, and other aspects making the disease difficult to recognize. Globally, but in Madagascar particularly, many techniques have been used, from phenotypic to genotypic methods. But most of these techniques did not have enough resolution to uncover its diversity until the advent of WGS that enabled the identification of genetic markers like VNTRs and SNPs leading to the cessation of the use of old techniques. Today, we have a clear idea, even if not exhaustive, of the distribution of the subgroups of *Y*. *pestis* in Madagascar, and even the story of the evolution and spread for some subgroups like the s subgroup. However, there are still some gaps in the study of the evolution of *Y*. *pestis* in Madagascar. The acquisition of foreign genetic materials through HGT, for example, is an area that has not been fully explored yet although it exists, according to previous studies in Madagascar but also in other countries. In conclusion, genotyping of *Y*. *pestis* from Madagascar has revealed important insights about plague that are relevant for better understanding this disease in Madagascar, but also the historical pandemics and the potential use of *Y*. *pestis* as a biological weapon. This highlights the importance of having genotyping capabilities available in highly endemic disease settings. Having these capabilities available in developing countries for surveillance and outbreak management is key to preparing for future epidemics and control of plague and other diseases [71].

**Key Learning Points**

- Genotyping *Y*. *pestis*, which is a clonal pathogen, has been challenging before the advent of high-resolution techniques such as WGS, MLVA, and SNPs genotyping.
- These high-resolution genotyping methods allowed different types of analysis to be run on Malagasy strains of *Y*. *pestis*, such as phylogenetic, investigation of transfer events, estimation of subpopulations emergence, and the study of the persistence of plague in rodent reservoirs and flea vectors.
- To date, the implications of horizontal gene transfer on the evolution and diversity of *Y*. *pestis* in Madagascar has not been investigated outside the study of antibiotic resistant strains.

**Key papers in the field**

1. Morelli G, Song Y, Mazzoni CJ, Eppinger M, Roumagnac P, Wagner DM, et al. *Yersinia pestis* genome sequencing identifies patterns of global phylogenetic diversity. Nat Genet. 2010;42:1140–3. doi: 10.1038/ng.705
2. Vogler AJ, Chan F, Wagner DM, Roumagnac P, Lee J, Nera R, et al. Phylogeography and molecular epidemiology of *Yersinia pestis* in Madagascar. PLoS Negl Trop Dis. 2011;5:e1319. doi: 10.1371/journal.pntd.0001319
3. Vogler AJ, Chan F, Nottingham R, Andersen G, Drees K, Beckstrom-Sternberg SM, et al. A decade of plague in Mahajanga, Madagascar: Insights into the global maritime spread of pandemic plague. MBio. 2013;4: e00623-12. doi: 10.1128/mbio.00623-12
4. Riehm JM, Projahn M, Vogler AJ, Rajerison M, Andersen G, Hall CM, et al. Diverse genotypes of *Yersinia pestis* caused plague in Madagascar in 2007. PLoS Negl Trop Dis. 2015;9: e0003844. doi:10.1371/journal.pntd
5. Vogler AJ, Andrianaivoarimanana V, Telfer S, Hall CM, Sahl JW, Hepp CM, et al. Temporal phylogeography of *Yersinia pestis* in Madagascar: Insights into the long-term maintenance of plague. PLoS Negl Trop Dis. 2017;11: e0005887. doi:10.1371/journal.pntd.0005887
